# Decomposing the Bragg glass and the peak effect in a Type-II superconductor

**DOI:** 10.1038/s41467-018-03267-z

**Published:** 2018-03-02

**Authors:** Rasmus Toft-Petersen, Asger B. Abrahamsen, Sandor Balog, Lionel Porcar, Mark Laver

**Affiliations:** 1 Department of Physics, Technical University of Denmark (DTU), DK-2800 Kongens Lyngby, Denmark; 2 Helmholtz-Zentrum Berlin für Materialien und Energie, Hahn-Meitner-Platz 1, D-14109 Berlin, Germany; 3 Department of Wind Energy, Technical University of Denmark (DTU), DK-4000 Roskilde, Denmark; 4 Adolphe Merkle Institute, University of Fribourg, Chemin des Verdiers 4, 1700 Fribourg, Switzerland; 5 Institut Laue-Langevin, 6 rue Jules Horowitz, 38042 Grenoble Cedex 9, France; 6 School of Metallurgy and Materials, University of Birmingham, Edgbaston, Birmingham B15 2TT UK

## Abstract

Adding impurities or defects destroys crystalline order. Occasionally, however, extraordinary behaviour emerges that cannot be explained by perturbing the ordered state. One example is the Kondo effect, where magnetic impurities in metals drastically alter the temperature dependence of resistivity. In Type-II superconductors, disorder generally works to pin vortices, giving zero resistivity below a critical current *j*_c_. However, peaks have been observed in the temperature and field dependences of *j*_c_. This peak effect is difficult to explain in terms of an ordered Abrikosov vortex lattice. Here we test the widespread paradigm that an order-disorder transition of the vortex ensemble drives the peak effect. Using neutron scattering to probe the vortex order in superconducting vanadium, we uncover an order-disorder transition from a quasi-long-range-ordered phase to a vortex glass. The peak effect, however, is found to lie at higher fields and temperatures, in a region where thermal fluctuations of individual vortices become significant.

## Abstract (web summary)

The disordering of the vortex lattice in a type-II superconductor is widely perceived to underpin unusual peaks in the temperature and field dependence of critical current. By contrast, here Toft-Petersen et al. find an order-disorder transition in a superconducting vanadium sample that is unconnected with peaks observed in critical current.

## Introduction

It has taken many decades to unravel the effect of weak disorder on the vortex lattice. In the 1970s, investigations initiated by the famous Russian theorist Anatoly Larkin found that any random disorder, no matter how weak, would destroy long-range order^1^. In Larkin’s perturbative approach^1,2^, vortex displacements grow linearly with vortex separation *r*, as measured by the displacement correlation function $b(r) = \left\langle {\left( {{\bf{u}}_j - {\bf{u}}_l} \right)^2} \right\rangle$, where **u**_*j*_ is the displacement of the *j*th vortex. This leads to an absence of divergent Bragg peaks. Yet resolution-limited Bragg peaks from vortex ensembles are clearly observed in scattering experiments^3–6^. It turns out the perturbative approach becomes invalid at in-plane spacings *r* = *r*_c_, where displacements become larger than *ξ*, the characteristic scale of the disorder potential. The behaviour at spacings *r* < *r*_c_ is accordingly described as belonging to the Larkin or random-force regime. At larger scales, vortices compete for minima in the disorder potential, slowing the algebraic growth of displacements to *b*(*r*) ∝ *r*^2*ζ*^, where the roughness exponent $\zeta < \frac{1}{2}$. In this so-called random manifold regime, theoretical values for *ζ* can be derived from the elastic Hamiltonian by scaling estimates^7,8^, renormalisation group analysis^9^ or variational replica-symmetry breaking techniques^10,11^. A summary of theoretical values for *ζ* is reproduced in Table 1.

**Table 1:** Properties of vortex–vortex correlations in superconducting vanadium

The topologically pristine Bragg glass phase is predicted by elastic theory with weak underlying disorder^11^. It has quasi-long-range positional order comprising of logarithmically growing displacements *b*(*r*) ∝ ln *r* and algebraically decaying translational correlations $c_{\mathrm{g}}(r) \propto r^{ - \eta _g}$ at large length scales *r* > *r*_A_. At smaller scales *r* < *r*_A_, correlations show the behaviour of a random manifold with *b*(*r*) ∝ *r*^2*ζ*^ and *c*_g_(*r*) ∝ exp[−(*r*/Λ_*g*_)^2*β*^]. Our observed exponents in the Bragg glass phase are compared with predicted values from elastic theory^9,11^. A slight dependence of these exponents on the elastic moduli is expected^9^ and here we list values appropriate for our vanadium sample. The Bragg glass is expected to become unstable at higher fields or temperatures. As shown by the data at 2.7 K and 0.17 T, the asymptotic regime is the first to be suppressed as the vortex ensemble disorders

At larger scales *r* = *r*_A_, where vortex displacements become of order $a_0 = \left( {2{\mathrm{\Phi }}_0{\mathrm{/}}\sqrt 3 B} \right)^{\frac{1}{2}}$, the lattice spacing set by flux quantisation, the periodicity of the system becomes crucial^12^. Here the random manifold gives way to a Bragg glass regime. This asymptotic regime at *r* > *r*_A_ is characterised by a slower, logarithmic growth^13^ *b*(*r*) ∝ ln *r* and a translational order correlation function $c_{\mathrm{g}}(r) = \left\langle {{\mathrm{e}}^{{\mathrm{i}}{\bf{g}}.\left( {{\bf{u}}_j - {\bf{u}}_l} \right)}} \right\rangle$ that decays algebraically with an exponent *η*_g_ (Table 1)^9,11^. The result is quasi-long-range order with algebraically diverging Bragg peaks and so the resulting vortex phase is referred to as the Bragg glass. Note that this term is used to describe both the asymptotic regime and, synecdochically, all three regimes in length scale. Where clarity is called for, we refer to the former as the Bragg glass regime and the latter as the Bragg glass picture.

The Bragg glass picture is expected to break down when dislocations become important. Upon increasing field or disorder strength, a transition to a short-range ordered vortex glass phase is expected as the pinning energy exceeds the plastic deformation energy^14–16^. Notionally, disorder affects translational order more than orientational order, so the vortex glass is likely hexatic^17^. The orientational order is characterised by the correlation function $g_6(r) = \left\langle {{\mathrm{e}}^{{\mathrm{i}}6\left( {\theta _j - \theta _l} \right)}} \right\rangle$, where *θ* is the nearest neighbour bond angle^17,18^. Experimentally *g*_6_(*r*) is observed to decay algebraically $\propto r^{ - \eta _6}$ in both the Bragg glass and vortex glass phases. A previous SANS study^19^ of the Bragg glass regime in niobium reported *η*_6_ = 0.07. In the vortex glass, images of disordered vortex ensembles on the surfaces of NbSe_2_^20^ and Bi_2_Sr_2_CaCu_2_O_8+*δ*_^21,22^ yield *η*_6_ ≈ 0.06–0.35. These images also show a much faster, exponential decay of translational order, consistent with a hexatic vortex glass.

At temperatures close to the upper critical field *B*_c2_(*T*) at which bulk superconductivity disappears, thermal fluctuations become increasingly important. They drive a proliferation of dislocations and a thermodynamic melting of the vortex lattice. The relative role of thermal fluctuations is quantified by the Ginzburg number^8^ ${\mathrm{Gi}} \approx \left( {\mu _0k_{\mathrm{B}}T_{\mathrm{c}}\kappa ^2{\mathrm{/}}2\xi ^3B_{{\mathrm{c2}}}^2} \right)^2$. The thermal melting line *B*_m_(*T*) of the vortex lattice can be estimated from the phenomenological Lindemann criterion $\left\langle {u^2} \right\rangle = c_{\mathrm{L}}^2a_0^2$ whereby melting occurs when displacements become a fraction *c*_L_ of the lattice spacing *a*_0_. Typically *c*_L_ ≈ 0.1–0.2. This gives^23,24^

1 $$\left( {B_{{\mathrm{c2}}}(T) - B_{\mathrm{m}}(T)} \right){\mathrm{/}}B_{{\mathrm{c2}}}(0) \approx 0.43c_{\mathrm{L}}^{ - \frac{4}{3}}{\mathrm{Gi}}^{\frac{1}{3}}t^{\frac{2}{3}}(1 - t^2)^{\frac{2}{3}}$$

where the reduced temperature *t* = *T*/*T*_c2_. Since Gi ∝ *ξ*^−6^, the position of the melting line is strongly dependent on the coherence length *ξ*. For cuprate superconductors, *ξ* is a few nanometres, so Gi ≈ 10^−2^–10^−3^ and the melting line is expected 0.1–10 K (depending on the field) below *B*_c2_(*T*). The expected position of the melting line is confirmed by experiments on the cuprates^25–27^. On the other hand, in low-*κ* superconductors the melting line is much more difficult to resolve^5,28^ due in part to the smallness of Gi. For our vanadium sample *κ* = 1.3, *ξ* = 26 nm (see Methods) and Gi ≈ 6 × 10^−10^, so *B*_m_(*T*) is anticipated to lie very close to *B*_c2_(*T*). For example, at 0.3 T and with *c*_L_ = 0.2, *B*_m_(*T*) is within 8 mK of *B*_c2_(*T*).

**Fig. 1:**
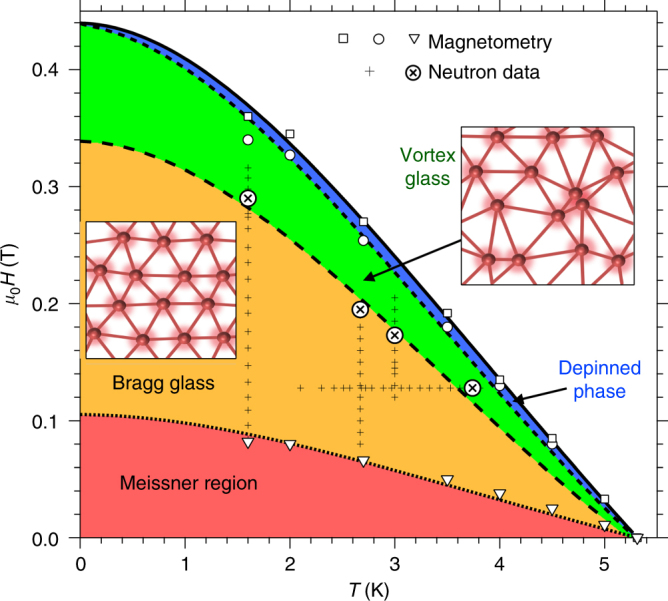
Phase diagram. Vortex phase diagram for our vanadium single crystal from magnetometry and neutron scattering experiments. Superconductivity appears below the upper critical field *B*_c2_(*T*) (squares and solid line), with vortices forming between *B*_c2_(*T*) and the lower critical field *B*_c1_(*T*) (triangles and dotted line). The critical current *j*_c_ is observed to collapse in a region close to *B*_c2_(*T*) (circles and short-dashed line). This collapse indicates that vortices are detaching from their pins. Vortex correlations are probed directly by neutron scattering. Plus signs mark the temperatures and fields where neutron scattering data were collected. The disappearance of neutron diffraction peaks (crosses in circles) marks the vortex order-disorder transition *H*_dis_(*T*). Short-dashed and long-dashed lines are fits based on different types of Lindemann criteria^23^ described in the text. The insets schematically illustrate the vortex phases either side of the order-disorder line. Intriguingly, the peak effect is not located at the order-disorder line. Instead it is observed close to the depinning line

Equation (1) is obtained with consideration of thermal fluctuations only. A Lindemann-like approach can also be used to predict the field and temperature dependence of the order-disorder transition line *B*_dis_(*T*) separating the Bragg glass and the vortex glass phases^23,29,30^. To do this, disorder-induced fluctuations must be considered. At *B*_dis_(*T*) the topologically ordered Bragg glass phase becomes unstable to the formation of dislocations. The dislocation network is anticipated^16^ to appear at scales ≈ *r*_A_ where displacements are on the order of *a*_0_. This can be shown to be equivalent to a generalised Lindemann criterion of the form $u^2(a_0,0) = c_{\mathrm{L}}^2a_0^2$ where displacements **u**(*r*, *l*) are now parameterised by the separations in and out of the vortex plane^29,30^. Note that the order-disorder transition can be driven entirely by disorder-induced displacements. Consequently as thermal fluctuations vanish for *T* → 0, *B*_dis_(0) remains distinct from and less than *B*_c2_(0) (Fig. 1), in contrast to the fluctuation-induced melting line *B*_m_(*T*), which curves up to meet *B*_c2_(0).

**Fig. 2:**
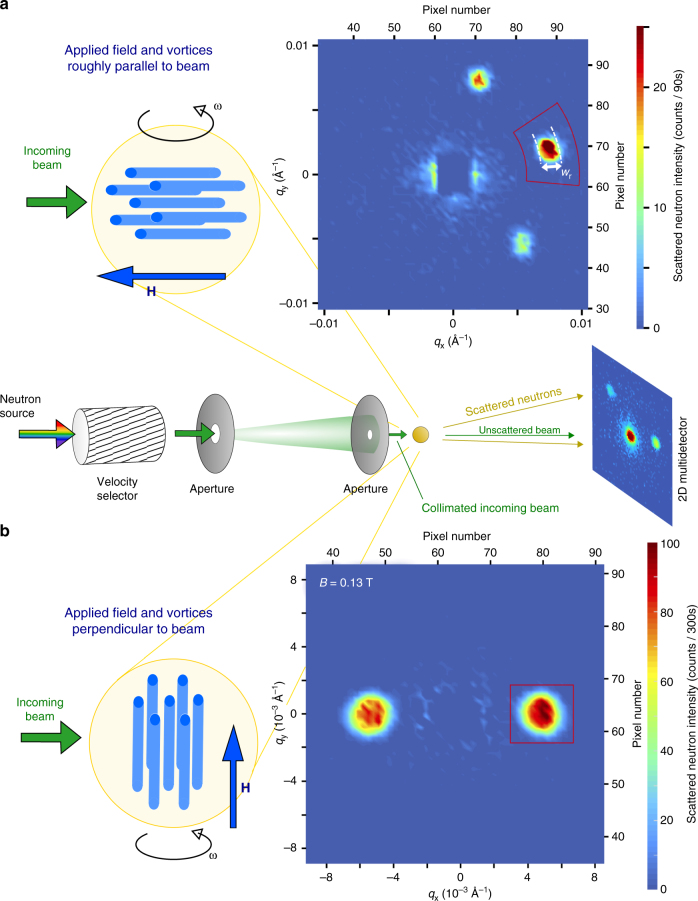
Neutron scattering geometries. Small-angle neutron scattering (SANS) instruments are usually pinhole cameras with small apertures to tightly collimate the incoming neutron beam. The magnetic field profile presented by vortices in the sample diffracts neutrons. Rocking curves are collected by rotating the sample, field and vortices together through the Bragg condition. Two experimental geometries may be used, exploiting the significantly better instrument resolution in the direction probed by the rocking curve: (**a**) The applied magnetic field and vortices are orientated roughly parallel to the neutron beam. Here the rocking curve is most sensitive to correlations along the vortices. A typical image of the 2D SANS multidetector at the peak of the rocking curve of the right Bragg spot is shown. The rotation axis for this rocking curve is indicated by *ω*. The unscattered neutron beam in the centre of the image at **q** = 0 is blocked by a beamstop. (**b**) The rocking curve probes correlations in the plane of the vortices when the applied magnetic field and vortices are orientated perpendicular to the neutron beam. The detector image shows the peak of the rocking curve of the right spot at 0.13 T

Here we use small-angle neutron scattering (SANS) to probe the long-range correlations of vortex ensembles in a vanadium single crystal. The SANS technique provided the first experimental evidence for the Bragg glass picture: a dependence of the diffracted peak intensity upon magnetic field *B* that could not be explained by perfect crystalline order^3^. We also find evidence from the field dependence of the diffracted intensity for a Bragg glass picture at intermediate fields in the phase diagram (Fig. 1). Furthermore we demonstrate the presence of a Bragg glass regime by characterising the shape of the diffraction peak in a high-resolution experimental set-up (Fig. 2) and using reverse Monte Carlo refinement to extract correlation functions from our data^19^. Our manuscript proceeds as follows: first we characterise the underlying disorder and the peak effect using magnetometry data on our vanadium single crystal. We subsequently examine our SANS data collected over several experiments and using different experimental setups. These SANS experiments allow the order-disorder transition to be located. Comparing the order-disorder transition line *B*_dis_(*T*) determined from SANS with the critical current density *j*_c_ determined by magnetometry, we discern no jump in *j*_c_ around *B*_dis_ in our sample. Instead, we observe a nascent peak effect at fields and temperatures close to *B*_c2_(*T*). This dissimilarity is at odds to the commonly held notion that the peak effect is underpinned by the order-disorder transition from Bragg glass to vortex glass.

## Results

### Critical current from magnetometry

**Fig. 3:**
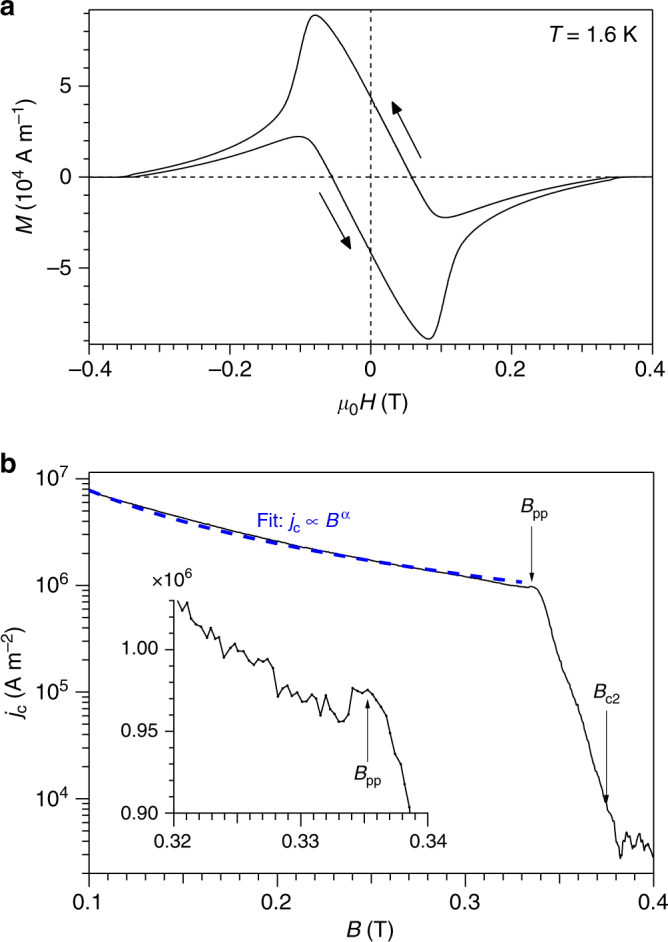
Magnetometry at 1.6 K. **a** *M*(*H*) loop for our vanadium single-crystal with $H \parallel [111]$. **b** Critical current density *j*_c_ calculated from the width Δ*M* of the magnetisation loop. *j*_c_(*B*) decreases monotonically except for a small peak, labelled *B*_pp_, observed just before the sharp downturn where thermal fluctuations start depinning vortices. The dashed line is a fit to an algebraic decay *j*_c_ ∝ *B*^*α*^. Fitted value of *α* = −1.7. Inset is a magnification of the region around *B*_pp_

Our sample is a vanadium single crystal of cylindrical shape, with length 10 mm and diameter 2*R* = 5 mm. The [111] cubic crystal direction is coincident with the cylindrical axis. We measured the isothermal magnetisation *M* as a function of magnetic field *H* applied parallel to [111], collecting *M*(*H*) curves at several temperatures. Figure 3a shows a typical *M*(*H*) loop, measured at *T* = 1.6 K. Extracting the upper critical field from these loops, we find *B*_c2_(*T*) is well described by the empirical relationship^31^ $B_{{\mathrm{c2}}}(t) = B_{{\mathrm{c2}}}(0)\left( {1 - t^2} \right){\mathrm{/}}\left( {1 + \frac{2}{3}t^{7/4}} \right)$ with *B*_c2_(0) = 0.440 T. Compared to pure vanadium where *T*_c_ = 5.47 K^32^, the small increase in *B*_c2_(*T*) and the small suppression of *T*_c_ = 5.31 K measured for our sample indicate that the underlying disorder is weak. We obtain a mean free path *l* = 48 nm (see Methods). From the *M*(*H*) loops, the critical current density *j*_c_ = 3Δ*M*/2*R* is calculated using the critical-state model of Bean^33^. *j*_c_ is seen to be small <10^7^ Am^−2^ (c.f. Fig. 3b) compared to the depairing current density $j_0 \approx H_{\mathrm{c}}{\mathrm{/}}\lambda \approx B_{{\mathrm{c2}}}$/$\left( {\sqrt 2 \kappa \lambda } \right) \approx 4 \times 10^{12}$ Am^−2^, consistent with weak pinning.

Underlying disorder constrains vortex displacements with a pinning force density *F*_p_ of magnitude *j*_c_*B*. Close to *B*_c2_(*T*), thermal fluctuations enable vortices to ride over the pinning potential and *F*_p_ is accordingly expected to decrease at high fields and temperatures. It follows that there must be a maximum in *F*_p_ as a function of *B*. Many models have been proposed to describe the field dependences of *F*_p_ and *j*_c_. Motivated by data on alloy superconductors with strong pinning, the first models estimated the average pinning force per vortex, accounting semi-empirically for the type and geometry of the pins^34–37^. For isotropic superconductors like vanadium, these models^37,38^ place the maximum in *F*_p_(*B*) at fields ≥ 0.2*B*_c2_. For our vanadium sample we find *F*_p_(*B*) does scale with *B*_c2_(*T*), but the maximum is located at 0.13*B*_c2_, i.e. at lower fields than expected and the observed *j*_c_(*B*) dependence is not accurately reproduced by these semi-empirical models^34–37^.

More recent models for *j*_c_(*B*) consider the contribution to pinning at different length scales^8^ and the dynamic effects of flux creep^39^. These models give various *j*_c_ behaviours depending on the regime of field and temperature. For example, the large bundle pinning regime is entered when pinning is weak and vortex length scales are large compared to the penetration depth *λ*. Here non-locality of the elastic constants can be neglected. In our low-*κ* superconductor, *λ* ≈ 35 nm is short. The minimum vortex lattice spacing *a*_0_ is 74 nm, the value at *B*_c2_(0), so vortex lattice length scales generally exceed *λ* and we expect to be in the large bundle regime in most of the mixed state. In this regime, *j*_c_ is theoretically predicted to follow^8^

2 $$j_{\mathrm{c}} \approx \frac{{j_0}}{{\kappa ^2}}\left( {\frac{{a_0}}{{l_{\mathrm{c}}}}} \right)^6$$

where *l*_c_ is the scale delimiting the Larkin regime parallel to the vortices. The $a_0^6$ dependence yields^8,39^ an algebraic decay of *j*_c_(*B*) ∝ *B*^−3^. As shown in Fig. 3b, we do find *j*_c_(*B*) can be described by an algebraic decay for 0.1 T < *B* < 0.33 T. With values of *j*_0_ and *j*_c_ for our sample, Eq. (2) gives *l*_*c*_ ≈ 10*a*_0_ over this field range. However the observed algebraic decay is slower than the *B*^−3^ predicted, with a reduced exponent *j*_c_(*B*) ∝ *B*^−1.7^.

Perhaps it is not too surprising that no literature model describes our *j*_c_ data precisely. All of the theoretical and empirical models described^8,34–39^ focus on high-*κ* superconductors. Our sample has low *κ* = 1.3 and a large part of the superconducting phase diagram is occupied by a Meissner regime (Fig. 1). In what follows we concentrate our analysis at high *B*, away from the Meissner regime.

For most of the mixed state, i.e. from *B* = 0.10 to 0.33 T at 1.6 K (Fig. 3b), the sample supports a finite critical current, indicating that vortices are pinned and frozen. At *B* = 0.34 T there is a sharp downturn in *j*_c_ and it falls rapidly up to *B*_c2_. This indicates vortices are depinning as thermal fluctuations take hold just below *B*_c2_. Just below the depinning region at *B* = 0.335 T, a nascent peak in *j*_c_ can be seen (Fig. 3b inset). This peak effect is less pronounced than in other reports^40^, which is to be expected given the weak underlying disorder in our sample. The location of the peak effect is consistent with previous transport measurements and magnetometry on niobium and vanadium under neutron irradiation^41–43^ and with transport measurements on niobium and Nb—Ta alloys under plastic deformation^44,45^. These systematic studies all show the same development of the peak effect: with increasing irradiation or deformation, the peak effect emerges first at high fields close to *B*_c2_ before developing into a larger peak that pushes to lower fields as the density of pinning centres increases^41,42^. The reverse effect, where the peak effect is reduced and pushed back up to high fields, is also observed when samples are annealed following neutron irradiation^43^.

### Order–disorder line from small-angle neutron scattering

Following the seminal study by Larkin and Ovchinnikov^2^ on the collective pinning of vortex ensembles, we might expect the jump in *j*_c_ to be associated with a loss of vortex order. Indeed, peak effects have commonly been linked with order-disorder transitions of vortex lattices in the literature^23,40,46,47^ To test this paradigm, we directly probe the vortex lattice order in our sample using SANS (Fig. 2).

**Fig. 4:**
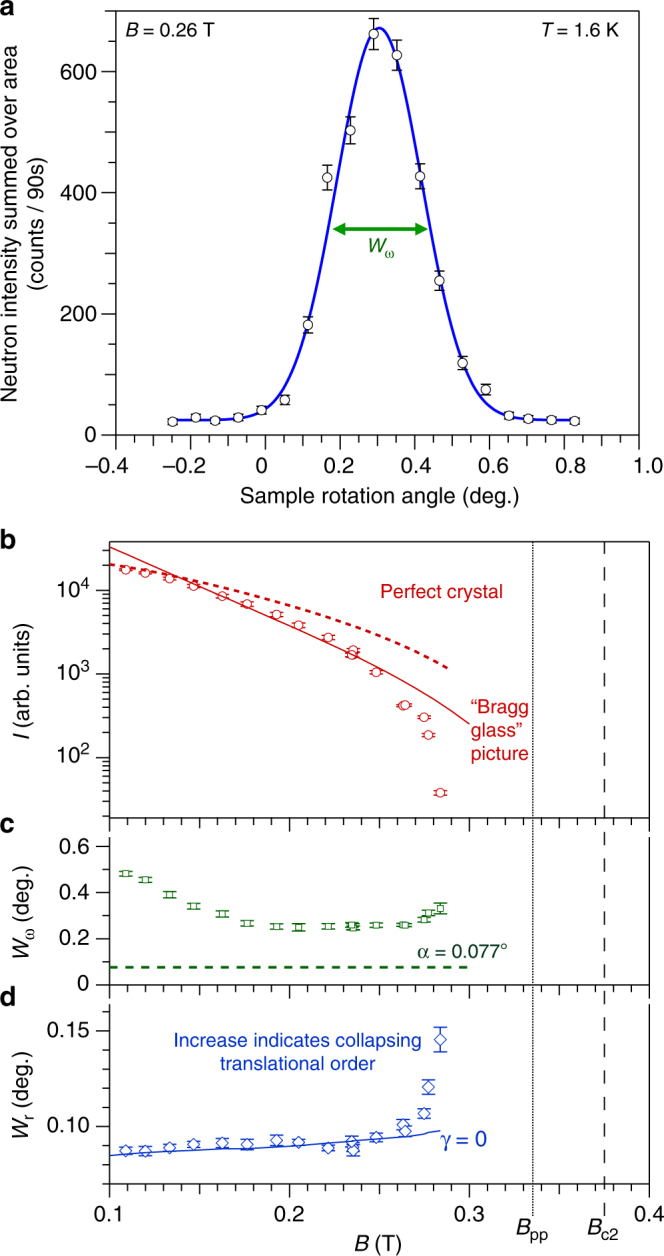
Neutron scattering in parallel field geometry at 1.6 K. **a** Rocking curves are collected by rotating the sample, field and vortices together through the Bragg condition. Neutron counts are summed over an area on the 2D multidetector encompassing the Bragg spot (red sector in Fig. 2a). Error bars are determined by Poisson statistics. Solid line is a Gaussian fit, with *W*_*ω*_ denoting the measured rocking curve width. **b** Field dependence, at *T* = 1.6 K, of the scattered neutron intensity *I *integrated over the rocking curve. Vortex ensembles are prepared by field-cooling, i.e. by cooling in the desired field from the normal state. Dotted line is a guide to the eye illustrating the form expected if vortices were arranged in a perfect 2D lattice. A steeper descent arises from the quasi-long-range order of the Bragg glass picture and scales to the data well (solid line) up to 0.26 T, whereupon *I *decays rapidly. Note that *I* vanishes much before the peak effect *B*_pp_. **c** *W*_*ω*_ probes correlations along the vortices in the parallel field geometry (Fig. 2a). The dominant contribution to the instrument resolution is the angular spread *a* of the incoming beam, illustrated by the dashed line. *a* = 0.077° corresponds to a maximum resolvable correlation length *s* = 2.35/(*ga*) = 240*a*_0_. **d** Radial width *W*_r_ of the Bragg spot on the 2D neutron multidetector at the peak of the rocking curve (Fig. 2a) is sensitive to correlations along the vortices and to translational correlations in the vortex plane. Solid line depicts the calculated radial width under the assumption of infinite translational correlational length 1/*γ*, i.e. it represents the contribution to *W*_r_ from instrument resolution and from finite correlations along the vortices. The rapid departure from this line at 0.27 T indicates collapsing translational order as the order-disorder transition is approached

Neutron scattering is sensitive to correlations between pairs of vortices via the structure factor $S = \mathop {\sum}\nolimits_{j,k} {\kern 1pt} {\mathrm{e}}^{ - {\mathrm{i}}{\bf{q}}.\left( {{\bf{r}}_j - {\bf{r}}_k} \right)}$ where **r**_*j*_ is the position of the *j*th vortex and **q** is the scattering vector. The measured scattered intensity as a function of *q* is the convolution of $\left| {h(q)} \right|^2S$ with the instrument resolution function, where *h*(*q*) is the form factor of a single vortex. For a perfect vortex lattice, the structure factor consists of *δ*-function Bragg peaks at reciprocal lattice vectors, with the first such vector appearing at $g = 2\pi \left( {2B{\mathrm{/\Phi }}_0\sqrt 3 } \right)^{\frac{1}{2}}$ in the case of a triangular lattice. Experimentally the Bragg peak intensity is quantified by integrating the measured scattering over three directions in reciprocal space. This is achieved by measuring rocking curves (Fig. 4a), where the sample, field and vortex lattice are rotated together through the Bragg condition. An area on the 2D SANS multidetector encompassing the Bragg spot is then summed and these summed counts, plotted versus rocking angle (Fig. 4a), are fitted to a Gaussian. The resulting integrated intensity *I* of the first order Bragg peak is shown in Fig. 4b, for *T* = 1.6 K after field-cooling. In the vortex glass, rapidly decaying translational order *c*_g_ is expected that does not support Bragg peaks. Combined with the rapid fall-off of the form factor^48^, this means little, if any, neutron scattering will be discernible from the vortex glass in our SANS experiments.

**Fig. 5:**
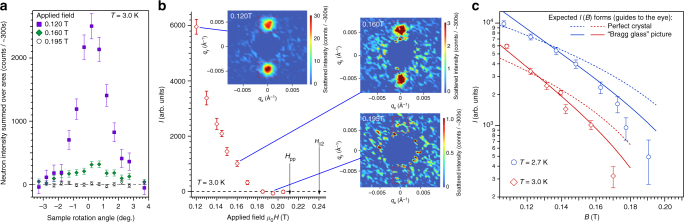
Field dependence of neutron scattering at higher temperatures. Field-cooled vortex ensembles are probed in perpendicular field geometry (Fig. 2b) with horizontal applied field. Backgrounds measured at zero field are subtracted. **a** Rocking curves at 3.0 K showing counts summed over an area on the 2D multidetector encompassing the top Bragg spot. Error bars are determined by Poisson statistics. **b** Top and bottom Bragg spot rocking curves are integrated numerically and the sum *I* plotted versus applied field. *I* vanishes much before the peak effect field *H*_pp_ anticipated from the phase diagram (Fig. 1). Each 2D image shows the SANS multidetector averaged over the rocking scan. Noise from subtraction at the centre of each image is masked. Red boxes indicate areas summed in the rocking curves. **c** Semilogarithmic plots at 2.7 and 3.0 K of *I* versus flux density *B* calculated from observed Bragg spot position *g* using the flux quantisation relation $g = 2\pi \left( {2B{\mathrm{/\Phi }}_0\sqrt 3 } \right)^{1/2}$ for a triangular lattice. Dotted lines are guides to the eye illustrating *I*(*B*) forms expected if vortices were arranged in perfect 2D lattices. Solid lines are guides to the eye showing the steeper descents expected in the Bragg glass picture

**Fig. 6:**
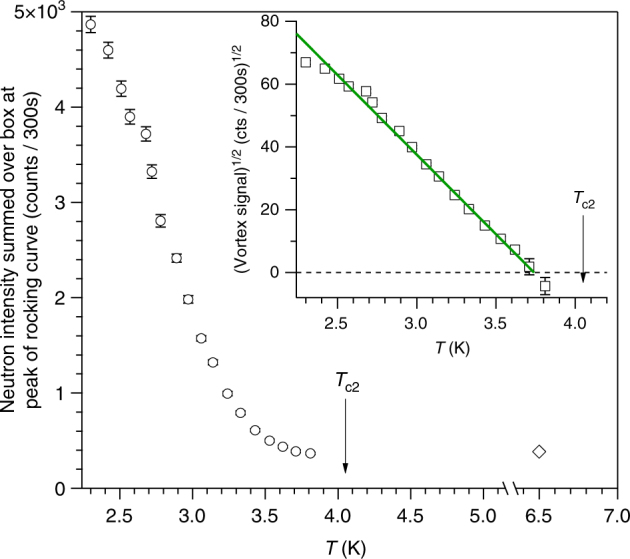
Temperature dependence at 0.13 T. Neutron scattering from the vortex ensemble is measured on warming, after field-cooling to 2 K. Using the perpendicular field geometry (Fig. 2b), neutron counts are collected at the peak of the rocking curve and summed over a box on the 2D multidetector encompassing the Bragg spot (red box in Fig. 2b). The diamond indicates a background measurement at *T* > *T*_*c*_ characterising the non-vortex scattering that arises predominantly from the walls of the closed-cycle refrigerator used to cool the sample. Error bars are determined by Poisson statistics. Inset shows the square root of the vortex signal *I*_p_, which is proportional to the vortex form factor *h* when the field is constant. Negative values of vortex signal, arising from the background subtraction, are indicated by plotting ${\mathrm{sgn}}\left( {I_{\mathrm{p}}} \right)\sqrt {\left| {I_{\mathrm{p}}} \right|}$. Green line is a guide to the eye showing a linear variation, as expected from mean field theory in the vicinity of a phase transition

We see in Fig. 4b that at *T* = 1.6 K the integrated intensity *I*(*B*) begins a downturn before quickly becoming indiscernible from the background at 0.29 T. We may therefore locate the vortex order-disorder line *B*_dis_(*T*) at 1.6 K, 0.29 T. Similarly we locate *B*_dis_(*T*) from the field dependence of *I* at two other temperatures (Fig. 5). All three *B*_dis_(*T*) points identified (crosses in circles in Fig. 1) lie well below *B*_c2_(*T*). *B*_dis_(*T*) may also be determined by measuring the scattered intensity as a function of temperature at constant field. In Fig. 6 we show the scattering measured at the peak of the rocking curve upon warming at 0.13 T, starting from a well-ordered vortex ensemble at 2 K. These data also demonstrate the vanishing of neutron intensity well below the upper critical field line and confirm that *B*_dis_(*T*) lies deep in the mixed state.

### Bragg glass picture from integrated neutron intensity

For a perfect crystalline lattice, integrating the structure factor *S* yields a factor 1/*g* at small scattering angles, so the field dependence of *I* is described by $\left| {h(g)} \right|^2{\mathrm{/}}g$. For the Bragg glass picture, on the other hand, the factor^3^ from integrating *S* depends on whether the SANS instrument resolution *s* is larger or smaller than the crossover scale between random manifold and Bragg glass regimes, at which displacements are of the order *a*_0_. For SANS measurements with the field and vortices aligned roughly parallel to the neutron beam, the width of the rocking curve probes correlations along the direction of the vortices (see Methods). Then if *s* < *l*_A_, where *l*_A_ is the crossover scale parallel to the vortices, integrating the structure factor in the Bragg glass picture yields a 1/*g*, i.e. a *B*^−1/2^ dependence similar to the perfect crystalline case. If *s* > *l*_A_, the Bragg glass picture produces an additional factor *B*^−3/2^. This hypothesis was borne out by the *I*(*B*) measured on (K,Ba)BiO_3_ in the first experimental report of the Bragg glass^3^. Similar *I*(*B*) Bragg glass dependences have been reported in the electron-doped cuprate Nd_1.85_Ce_0.15_CuO_4_ and in underdoped La_2−*x*_Sr_*x*_CuO_4_ (*x* < 0.15)^49^.

In Fig. 4b we compare the integrated intensity *I*(*B*) measured at 1.6 K with the field dependence expected for a perfect crystalline lattice and that expected in the Bragg glass picture. For these measurements, the instrument resolution *s* ≈ 240*a*_0_ along the direction of the vortices (see Methods). Up to *B* ≈ 0.26 T, i.e. before *I* falls-off more sharply as *B*_dis_ is approached, it can be seen that the field dependence in the Bragg glass picture describes the data rather better than for the perfect crystalline case. We deduce that *l*_A_ < *s* ≈ 240*a*_0_ from our experiments. In elastic theory^8^, length scales along the vortices and length scales in the vortex plane are coupled through the elastic moduli via $\sqrt {c_{44}{\mathrm{/}}c_{66}}$. Approximate expressions for the tilt modulus *c*_44_ ≈ *BH* and for the shear modulus are^50^

3 $$c_{66} \approx \frac{{B{\mathrm{\Phi }}_0}}{{16\pi \lambda ^2\mu _0}}\left( {1 - \frac{1}{{2\kappa ^2}}} \right)\left( {1 - \frac{B}{{B_{{\mathrm{c2}}}}}} \right)^2$$

The shear modulus *c*_66_ softens close to the upper critical field line as vortex cores start to overlap. Deeper in the mixed state, e.g. for fields 0.1 T < *B* < 0.33 T, we find the calculated aspect ratio $\sqrt {c_{44}{\mathrm{/}}c_{66}}$ lies in the range from 2 to 30. An upper bound for the crossover scale *r*_A_ in the plane of the vortices can thus be determined e.g. at 0.23 T, this upper bound is $s{\mathrm{/}}\sqrt {c_{44}{\mathrm{/}}c_{66}} \approx 30a_0$. In the next section, we see *r*_A_ can be precisely quantified using a high-resolution SANS setup.

### Bragg glass regime from reverse Monte Carlo refinement

**Fig. 7:**
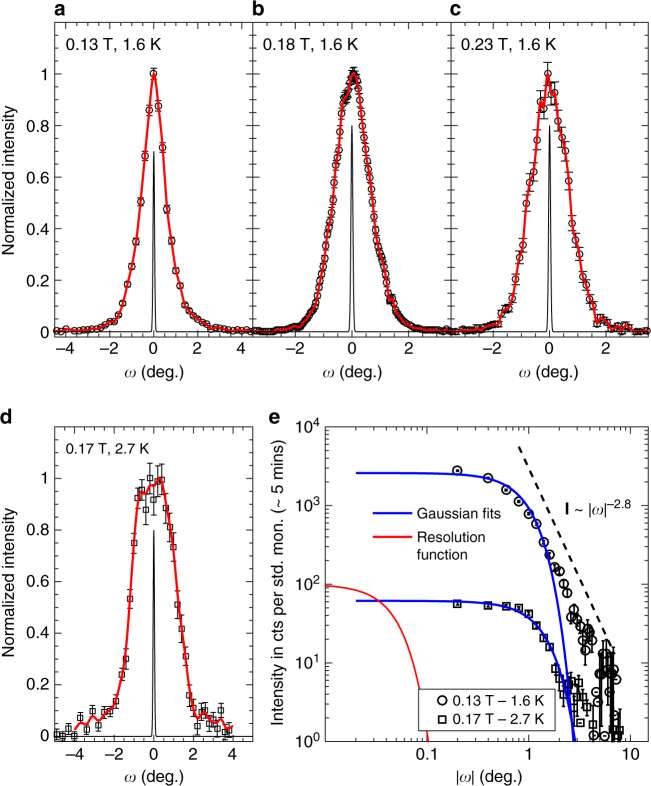
Neutron scattering in perpendicular field geometry. In the perpendicular field geometry (Fig. 2b), rocking curves probe correlations in the plane of the vortices. **a**–**d** Rocking curves of the first order diffraction peak at four selected points in the phase diagram, collected with long counting times to afford high quality data for reverse Monte Carlo refinement. Backgrounds measured at zero field have been subtracted. Ordinate axes are normalised for comparison. In reverse Monte Carlo refinement, simulated vortices are displaced using a Monte Carlo method until the experimental rocking curve (circles) is reproduced (red lines). Black lines indicate instrument resolution. **e** Rocking curves of **a** and **d** on logarithmic axes. Dashed line is a guide showing an algebraic decay. Algebraic tails are expected from the Bragg glass picture. Gaussian fits (blue lines) are drawn for comparison

To gain more detailed information about the Bragg glass and vortex glass phases in the sample, we employ a second experiment geometry in our SANS measurements. The SANS data in Fig. 4 are collected in the first experiment geometry where applied field and vortices are orientated roughly parallel to the incoming neutron beam (Fig. 2a). In the second experiment geometry, the applied field and vortices are orientated perpendicular to the beam (Fig. 2b). In this geometry, a high instrument resolution in the plane of the vortices is achieved (see Methods) and rocking curves, collected by rotating the sample, field and vortex ensemble together through the Bragg condition, probe correlations within the vortex plane, transverse to the reciprocal lattice vector (Fig. 7). Such data (Fig. 7e) are seen to exhibit an algebraic decay with rotation angle *ω*. This algebraic decay is characteristic of the Bragg glass regime^11,19^. The observed exponent ≈ −2.8 (Fig. 7e) is roughly consistent with the value ≈ *η*_g_ − 3 predicted^11^ for the Bragg glass regime where *c*_g_(*r*) decays algebraically $\propto r^{ - \eta _{\mathrm{g}}}$, with *η*_g_ ≈ 1 (Table 1).

To uncover more information about the vortex correlations, we use the reverse Monte Carlo (RMC) technique to refine the peak shapes of rocking curves collected in the perpendicular field geometry^19^. Up to 220,000 vortices are simulated on a computer, with their positions modified successively via Monte Carlo updates, until the observed peak shape is reproduced (see Methods). Meaningful refinements require experimental data that span several decades of rotation angle *ω*. Long data collection times are needed for the tails where the scattering is weak. We measured such datasets at four selected fields and temperatures in the phase diagram (Fig. 7).

**Fig. 8:**
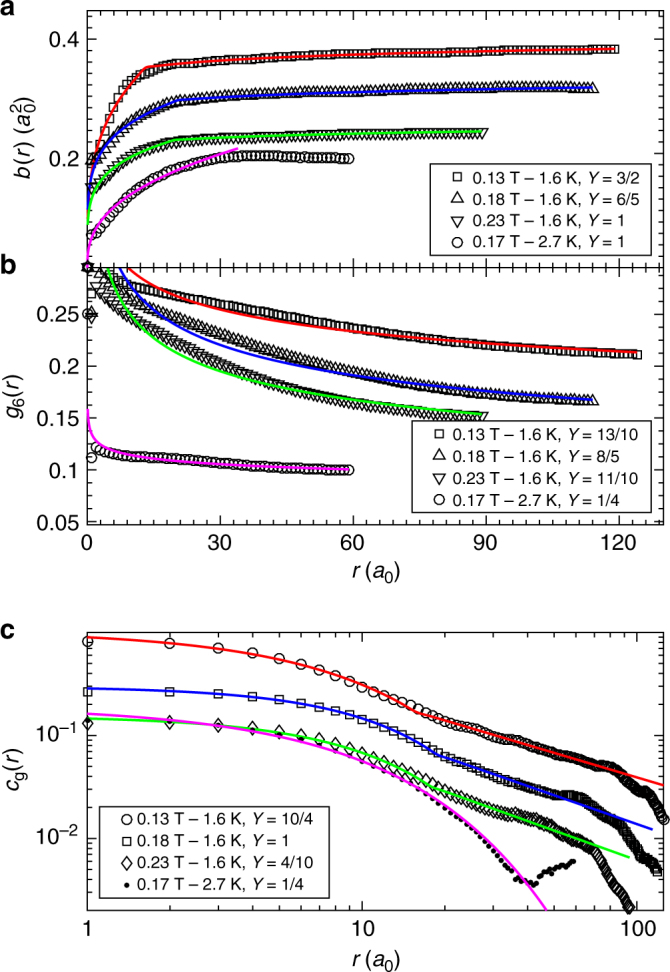
In-plane correlations educed by reverse Monte Carlo refinement. Correlation functions calculated from simulated vortex ensembles (open symbols) that reproduce the neutron scattering data in Fig. 7. For clarity curves are offset with a multiplication factor *Y*. (**a**) Displacement correlator $b(r) = \left\langle {\left( {{\bf{u}}_j - {\bf{u}}_l} \right)^2} \right\rangle$. (**b**) Hexatic orientational order $g_6(r) = \left\langle {{\mathrm{e}}^{{\mathrm{i}}6\left( {\theta _j - \theta _l} \right)}} \right\rangle$ is fitted with a single algebraic decay $\propto r^{ - \eta _6}$. (**c**) Translational order $c_{\mathrm{g}}(r) = \left\langle {{\mathrm{e}}^{{\mathrm{i}}{\bf{g}}.\left( {{\bf{u}}_j - {\bf{u}}_l} \right)}} \right\rangle$ and *b*(*r*) comprise three regimes in the Bragg glass picture: the Larkin, random manifold and Bragg glass regimes (see text). Here the Larkin regime lies at indiscernibly small length scales and two-part functions fit *b*(*r*) and *c*_g_(*r*) (solid lines) for the three datasets at 1.6 K. For the 0.17 T, 2.7 K dataset, which lies close to the order-disorder line, a single function fits *c*_g_(*r*) corresponding to the random manifold regime

Once the simulated rocking curve numerically reproduces the experimentally measured rocking curve (Fig. 7), correlation functions may be calculated from the simulated vortex positions. A simulated vortex ensemble is not unique: there are many possible simulated microstates that can reproduce the experimental peak shape. However, similar correlation functions are observed to result from such microstates^19^. In Fig. 8 we show typical correlation functions extracted using RMC at each of the four points measured in the phase diagram. Looking first at the three datasets collected at *T* = 1.6 K and *B* = 0.13, 0.18 and 0.23 T, we see these show the same qualitative features, namely two distinct regimes in length scale. At small *r*, the displacement correlation function *b*(*r*) is seen to increase algebraically while *c*_g_(*r*) is seen to decay as a stretched exponential. This clearly represents a random manifold regime^9–11^ and accordingly here we fit the data to *b*(*r*) ∝ *r*^2*ζ*^ and *c*_g_(*r*) ∝ exp{−(*r*/Λ_g_)^2*β*^}. The fitted values of roughness exponent *ζ*, exponential decay exponent *β* and the effective correlation length Λ_g_ agree well with theoretical predictions (Table 1). The absence of an observable Larkin regime at small *r* is consistent with the weak disorder of our sample^11^. Using the above estimate of *l*_c_ ≈ 10*a*_0_ from the critical current, the Larkin regime would be expected at in-plane length scales below $r_{\mathrm{c}} \approx l_{\mathrm{c}}{\mathrm{/}}\sqrt {c_{44}{\mathrm{/}}c_{66}} \approx 2a_0$, a value which is too small to be resolvable from the *r*-dependence of correlation functions.

At large *r*, we identify a Bragg glass regime (Fig. 8). In this asymptotic regime, *b*(*r*) ∝ ln *r* and $c_{\mathrm{g}}(r) \propto r^{ - \eta _{\mathrm{g}}}$, with the fitted *η*_g_ ≈ 1 (Table 1). The orientational order *g*_6_(*r*) follows an algebraic decay $\propto r^{ - \eta _6}$ through both the random manifold and the Bragg glass regimes. The fitted exponent *η*_6_ ≈ 0.2 (Table 1) is rather less than the translational exponent *η*_g_, reflecting the longer range of bond orientational order compared to translational order. We conclude that a Bragg glass phase is present at fields and temperatures below the order-disorder transition *B*_dis_(*T*).

### Fracturing of the vortex lattice

We turn now to the fourth dataset collected with long counting times in the perpendicular geometry at 0.17 T and 2.7 K, a point which lies close to the order-disorder transition *B*_dis_(*T*) (c.f. Fig. 1). Here the rocking curve exhibits a distinctly different shape to the other three RMC datasets measured deeper in the Bragg glass phase. Its brow appears broader (Fig. 7d) and its tails seem less algebraic (Fig. 7e). The correlation functions extracted using RMC show a concomitant suppression of the asymptotic Bragg glass regime: the algebraic decay at large *r* in *c*_g_(*r*) is no longer visible (Fig. 8c). Only a stretched exponential decay remains, with fitted exponents indicative of the random manifold regime (Table 1). *b*(*r*) also shows the sublinear growth expected in this regime.

There is more intriguing behaviour at large *r*. Here, instead of the slow logarithmic growth that would signal a Bragg glass, *b*(*r*) is seen to saturate at ≈ 30*a*_0_ (Fig. 8a). This suggests the vortex ensemble is fracturing into domains, with reasonable positional order being maintained within each domain and large jumps in displacement, with possible locally amorphous regions, at the domain walls. Indeed, a finite crystalline domain of dimension ≈ 30*a*_0_ would produce a Bragg peak of finite width ≈ 1.6°, which is roughly consistent with the broadening observed in the rocking curve (Fig. 7d). The orientational order *g*_6_(*r*) for this RMC dataset is reduced compared to the other three RMC datasets, but it continues to decay slowly, with a fitted algebraic exponent *η*_6_ = 0.07. Moreover, *g*_6_(*r*) persists beyond the domain length scale ≈ 30*a*_0_, indicating that domains share roughly similar nearest neighbour directions.

The fracturing observed of the Bragg glass is evocative of the prediction of a multidomain glass phase, that should separate the Bragg glass and vortex liquid phases^47^. There are differences between prediction and experiment, however. The predicted multidomain glass phase should be confined to a thin sliver in the phase diagram coincident with the peak effect^47^. Here, on the other hand, the fracturing of the ordered phase is observed at much lower fields and temperatures than the peak effect. The predicted multidomain glass also has both orientational and translational order decaying rapidly beyond the domain length scale, but here we see the orientational order *g*_6_(*r*) survives to larger length scales even in the fractured vortex ensemble. Interestingly, qualitatively similar correlation functions were measured by decoration experiments on the high-*T*_c_ layered cuprate Bi_2_Sr_2_CaCu_2_O_8+*x*_ (BSCCO)^51^. The formation of domains was proposed to be a nonequilibrium effect due to finite cooling rates^51^. We have initiated further SANS explorations to clarify this.

## Discussion

The peak effect and other features in magnetometry and transport data are frequently assumed to be underpinned by changes in the positional order of the vortex lattice. Under this assumption, conclusions are sometimes drawn as to the nature of vortex phases without directly probing them. Several features can be identified in magnetometry or transport data, including the irreversibility line (*H*_irr_), the peak field (*H*_pp_) and the onset of the peak effect regime (*H*_po_). In the high-*T*_*c*_ cuprates, a broad peak in Δ*M* may also be present at low temperatures and fields deep in the mixed state and is accordingly designated the fishtail effect or second magnetisation peak^52^. There are reports of both a sharp peak effect close to *B*_c2_ and a broad second magnetisation peak well below *B*_c2_ being observed in the same sample^53,54^ indicating that these two effects are distinct. Accordingly, we should add to our list of features the onset field (*H*_fo_), the fishtail peak field (*H*_fp_) and the field at which the magnetisation shows a kink (*H*_fk_).

On the other hand, the route from ordered vortex ensemble to vortex liquid is envisaged to occur in only one or two sharp steps, i.e. a direct melting from Bragg glass to vortex liquid, or an order-disorder transition to a vortex glass followed by melting to the liquid. Recent scanning tunnelling microscope (STM) experiments on Co-doped NbSe_2_ indicate that there may be two vortex glass phases—a hexatic vortex glass and an amorphous vortex glass^55^—though it is not clear whether the two phases identified are truly distinct. Nonetheless, there are far fewer disordering transitions than there are features in Δ*M* and *j*_c_. It is an open question as to which of these features, if any, should be tied to an underlying order-disorder transition in the vortex ensemble. The irreversibility line may reasonably be associated with the melting transition^25^, since a vortex liquid phase cannot ordinarily support a finite critical current. To which feature, on the other hand, should we associate the Bragg glass to vortex glass transition?

To date only a handful of studies have directly probed the order of the vortex ensemble and simultaneously pinpointed features in magnetisation or critical current. The first such study found the SANS intensity disappeared at the irreversibility line in BSCCO^25^. This occurred at temperatures consistent with estimates of the melting line using the Ginzburg number and the Lindemann criterion, signalling a link between vortex lattice melting and *H*_irr_. In addition, the diffracted neutron intensity was found to vanish abruptly with increasing fields at temperatures well below the irreversibility line, with no reported corresponding features in the bulk magnetisation^25^. A subsequent muon spin rotation study indicated that this vanishing was due to a 3D to 2D crossover, where the pancake vortices in BSCCO, which arise due to its highly anisotropic layered structure, become decoupled between the superconducting layers^56^.

Subsequent studies have focussed on the quasi-two-dimensional layered 2H-NbSe_2_ system. Here, due to coexisting charge order, the vortex cores are strongly anisotropic in the plane, adopting a sixfold star shape^57,58^. Magnetic impurities in 2H-NbSe_2_ are adorned by bound states with the same sixfold star shape^59^ which, we suggest, may drive the additional transition from hexatic glass to an amorphous vortex glass observed recently in Co-doped NbSe_2_ samples^55^. Interestingly this transition appears to coincide with the peak field *H*_pp_(*T*) in these samples^55^, indicating that the Bragg glass to hexatic glass transition should lie at lower fields and temperatures, e.g. perhaps at the onset *H*_po_(*T*). This scenario is consistent with a recent SANS study^6^ on clean NbSe_2_ where the SANS intensity all but disappears at *H*_po_(*T*).

Unfortunately the experimental picture is neither so simple nor general. In Fe-doped NbSe_2_, a well-ordered vortex lattice can be observed by SANS at fields much higher than *H*_pp_, leading to the conclusion that the peak effect is unrelated to a bulk order–disorder transition^60^. This conclusion is shared by an earlier decoration study on clean and Fe-doped NbSe_2_^61^. The picture is muddied further by studies on isotropic conventional superconductors, like niobium, vanadium and V_3_Si, where vortices have the usual line structure. In V_3_Si^62^, the diffracted SANS intensity disappears at *H*_pp_(*T*) but in Nb there are conflicting reports: in one SANS study *H*_pp_(*T*) appears to coincide with the loss of in-plane positional order^63^ but in another study a clear SANS signal indicating good vortex order is observed above the peak effect^4^. Together with our observation of a Bragg glass to vortex glass transition lying at much lower fields and temperatures than *H*_pp_(*T*), we are drawn to the inevitable conclusion that the origin of the order–disorder transition and the origin of the peak effect are not a priori the same.

Recent experimental investigations also show that metastability of the vortex configuration and the associated dependences on history affect the vortex order and consequently the perceived position of the order–disorder transition^4,6,55,62^. Bulk characteristics from transport^46,62^ and magnetic susceptibility^6^ are also similarly affected. Here we note that our data point for *B*_dis_(*T*) at 0.13 T and 3.74 K (Fig. 6) is obtained by warming the sample at constant field, so it is possible that the vortex configuration is superheated at the perceived order–disorder transition temperature of 3.74 K. If this were the case however, the equilibrium order–disorder transition would lie at even lower temperatures than we report here. Our conclusion, that the order–disorder transition and the peak effect are not necessarily related, would abide.

What are the origins of the order–disorder transition and of the peak effect? One possibility is a change in the nature of the underlying pinning such as a crossover from weak collective to strong pinning^64^. For weak pinning, the pinning force density $F_{\mathrm{p}} = j_{\mathrm{c}}B = \left[ {f_{\mathrm{p}}^2{\kern 1pt} n_{\mathrm{p}}\left( {\xi {\mathrm{/}}a_0} \right)^2{\mathrm{/}}V_{\mathrm{c}}} \right]^{1/2}$, where *f*_p_ is the elementary pinning force and *n*_p_ = 1/*ξ*^2^*l* is the density of pins, $V_{\mathrm{c}} = l_{\mathrm{c}}r_{\mathrm{c}}^2$ is the correlation volume over which displacements reach the superconducting coherence length *ξ* following the random force model^2,64^. Strong and/or single pinning is identified when *f*_p_ overcomes the Labusch force^64^ $f_{\mathrm{L}} \approx {\mathrm{\Phi }}_0^2{\mathrm{/}}4\pi \mu _0\lambda ^2 \approx 10^{ - 11}$ N for our sample. Since our data do not provide a direct measure of *l*_c_ or *r*_c_, we make an overestimate of $V_{\mathrm{c}} \approx \sqrt {c_{44}{\mathrm{/}}c_{66}} {\kern 1pt} r_{\mathrm{A}}^3$ to yield an upper bound for *f*_p_, which is maximal at low inductions, reaching ≈10^−13^ N. Thus throughout the mixed state $f_{\mathrm{p}} \ll f_{\mathrm{L}}$ and weak collective pinning is effective. There is no change in pinning regime at *B*_dis_(*T*) or at the peak effect in our sample.

We return to the effect of thermal fluctuations. These allow the vortices to ride over the pinning potential and result in a collapse of the critical current. The onset of fluctuations in our sample is marked by the sharp downturn in *j*_c_ close to the upper critical field. At *T* = 1.6 K, this depinning of vortices occurs at *B* = 0.34 T (Fig. 3b). At 0.3 T, the depinning line lies ≈ 140 mK below *B*_c2_(*T*), substantially below the thermally driven vortex lattice melting line, which we recall is 8 mK below *B*_c2_(*T*) from Eq. (1). One can quantify how far a (*B*, *T*) point in the phase diagram lies from *B*_c2_(*T*) using the scaled Thouless temperature *a*_T_ from the lowest Landau level of the Ginzburg-Landau theory. Thermodynamic melting is expected^65^ at *a*_T_ = −9.5. Isotherms of constant *a*_T_ have approximately the same form as the melting line *B*_m_(*T*) from Eq. (1), so we may identify $- a_{\mathrm{T}} \approx 0.43c_{\mathrm{L}}^{ - \frac{4}{3}}$, e.g. *a*_T_ = −9.5 corresponds to a Lindemann number *c*_L_ ≈ 0.1. The depinning line in our sample corresponds to *a*_T_ ≈ −60 or *c*_L_ ≈ 0.02. The peak effect lies just below this line, i.e. on the edge of the regime where thermal fluctuations dominate. The possibility that thermal fluctuations induce the peak effect has been pointed out previously^66,67^, however this view must be reconciled with the reported observations of well-ordered vortex lattices at temperatures and fields above the peak effect^4,60^. We suggest that thermal fluctuations sufficiently reduce the order parameter Ψ, such that weak underlying pinning is suddenly accommodated by a rapid but local change in the structure of the vortex cores at the peak effect. This instability, localised to the cores, is not significant at larger scales such as *λ* or *a*_0_ so lattice order is not necessarily disturbed through the peak effect.

At the order–disorder transition, on the other hand, the role of thermal fluctuations is insignificant. The four data points for *B*_dis_(*T*) (Fig. 1) lie close to the *c*_L_ = 0.006 or *a*_T_ = −360 isotherm, i.e. far from the fluctuation dominated regime. Disorder can be incorporated into lowest Landau level theory via random components in the $\left| {\mathrm{\Psi }} \right|^2$ term (*δT*_*c*_ pinning)^65^ and in the $\left| {\mathrm{\Psi }} \right|^4$ term^68^, yielding order–disorder lines where the value of *a*_T_ varies along the line. We do not find these provide sensible fits to our four data points. We do find a sensible fit is provided by the order–disorder line derived for *δT*_*c*_ pinning using a Lindemann-like approach^23^. In the limit of vanishing thermal fluctuations, i.e. Gi → 0, *B*_dis_(*T*) then takes the form^23^

4 $$1 - \frac{{B_{{\mathrm{dis}}}(T)}}{{B_{{\mathrm{c2}}}(T)}} \approx \left( {\frac{{2\pi }}{{c_{\mathrm{L}}^4}}} \right)^{\frac{1}{3}}D^2\left( {1 - t^2} \right)^{ - \frac{1}{3}}$$

*D* measures the strength of the disorder and is equal to *ξ*/*l*_c_ at 0 K in the Larkin model^23^. We fit this parameter (Fig. 1), taking *c*_L_ = 0.2 and obtaining *D* = 0.12, which is a reasonable value for weak pinning. This yields *l*_c_ ≈ 3 *a*_0_ at 0 K, consistent with our earlier estimate from *j*_c_ and with our RMC results. As *B*_dis_(*T*) is approached upon warming (Fig. 6), the SANS intensity falls continuously to zero, indicating that *B*_dis_(*T*) is a thermodynamic phase transition from Bragg glass to vortex glass. The radial width of the Bragg diffraction spot on the 2D SANS detector also increases as *B*_dis_ is approached (Fig. 4d), signalling a collapsing translational correlation length. We infer that continuous translational symmetry is broken at the Bragg glass to vortex glass transition. It is still a matter of debate, however, as to which symmetry—if any—is broken between the depinned vortex liquid and pinned vortex glass phases. We expect an exciting era in vortex matter physics, where intriguing possibilities such as the vortex glass being no more than a pinned, hexatic liquid^18^ are experimentally explored using increasingly available high-quality STM apparatus.

Overall, our data show that the peak effect and similar features in magnetometry or transport data may not a priori be due to a vortex order–disorder transition. SANS provides a direct probe of vortex order and reveals the order–disorder transition *B*_dis_(*T*) in our sample. It is mediated only by the weak underlying disorder, lying deep in the mixed state, far from the regime dominated by thermal fluctuations. A jump in *j*_c_ around *B*_dis_, as might be expected following the theory of Larkin and Ovchinnikov^2^, cannot be detected in our *j*_c_ data derived from magnetometry (Fig. 3b). In contrast, these data show a nascent peak effect at high temperatures and fields, where thermal fluctuations become apparent.

## Methods

### Laboratory characterisation

Bulk magnetic measurements were carried out using a high-field cryogen free measurement system (CFMS) at the DTU Risø Campus. The field was applied parallel to the [111] crystal axis. We used the Goodman-Gor’kov relations^69^ to compare the upper critical field *B*_c2_(*T*) and superconducting critical temperature *T*_c_ of our sample to the values reported by ref.^32^ for varying sample purity. We calculate a mean free path *l* = 48 nm, impurity parameter *α* = 0.84, superconducting coherence length *ξ*_0_ = 26 nm and London penetration depth *λ*(0) = 35 nm implying *κ* = 1.3 for our sample.

### Neutron scattering

SANS experiments were performed on three instruments: D22 at the Institut Laue-Langevin (Fig. 4), NG7 at the NIST Center for Neutron Research (Fig. 6) and, SANS-II at the Swiss Spallation Neutron Source (Figs. 5, 7 and 8). In a typical setup on D22, neutrons of wavelength *λ* = 0.9 nm with spread Δ*λ*/*λ* = 0.1 were collimated over a 18 m distance, providing a beam of angular spread *a* = 0.077°. Scattered neutrons were detected using a 2D multidetector placed 18 m behind the sample (Fig. 2). NG7 and D22 are at reactor sources where the neutron flux at the sample position is constant over experiment time scales. SANS-II is at a continuous spallation source with varying neutron flux, so in these experiments multidetector count rates are normalised with a monitor detector situated upstream of the sample. Rather than plotting detector counts/monitor counts, in Fig. 5a and in Fig. 7e the ordinates are scaled to a typical monitor value that was used for measurements containing low or zero vortex signal. When the spallation source is stable, this corresponds to a measurement time of 5 min per rocking angle.

The [111] axis of our vanadium crystal was aligned to within 0.2° of the applied magnetic field direction. Unless otherwise noted, vortex ensembles were prepared by cooling in the desired magnetic field from the normal state, i.e. field-cooled. Any misalignment between the applied field and the vortex directions due to crystalline anisotropy would have been much smaller than the observed peak widths. In niobium, which has a larger crystalline anisotropy than vanadium, 0.2° of misalignment from [111] would distort the vortex direction by 0.007°^70^. The edges of the sample were masked from the incident neutrons in view of possible demagnetisation effects.

Two experimental geometries were used in our SANS measurements: (a) the magnetic field was applied roughly parallel to the incident neutron beam (Fig. 2a); (b) the field was applied perpendicular to the beam (Fig. 2b). The parallel geometry was used with *a* = 0.077° on D22 to survey the field dependence (Fig. 4). This geometry also confirmed the absence of transitions in vortex lattice shape away from the hexagonal symmetry expected for fields along [111]. The perpendicular geometry was used on NG7 with vertical applied field (Fig. 2b) for the temperature dependence (Fig. 6). It was also used on SANS-II with horizontal applied field to further map the phase diagram (Fig. 5) and to collect rocking curves with high resolution (*a* = 0.035°) (Fig. 7) suitable for educing in-plane correlations by reverse Monte Carlo refinement. To describe the field dependences of the intensity *I* integrated over the rocking curve (Figs. 4b and 5c), we used the form factor *h* calculated for a hexagonal vortex lattice^48^.

As an initial approximation, the instrument resolution and mosaic spread of the vortex ensemble may be modelled as Gaussian distributions. All widths quoted in this manuscript are full-width half maxima. In either experiment geometry, the measured rocking curve width *W*_*ω*_ (Fig. 4a) is given by^71^

5 $$W_\omega ^2 = a^2 + c^2 + \sigma ^2$$

where *c* is the combined width parallel to the scattering vector: $c^2{\mathrm{/}}\theta _{\mathrm{B}}^2 = ({\mathrm{\Delta }}\lambda {\mathrm{/}}\lambda )^2 + \gamma ^2$ with *θ*_B_ the Bragg angle and *γ* the mosaic spread of the vortex ensemble parallel to the reciprocal lattice vector **g**. In the parallel geometry, *σ* probes correlations along the vortices. In the perpendicular geometry, *σ* probes the direction perpendicular to both the field and to **g**. The scattering angles are small, e.g. *θ*_B_ = 0.2° for 0.9 nm neutrons when *B* = 0.1 T, so *c* is small in Eq. (5) and the angular spread *a* of the incoming beam sets the minimum measurable width of the rocking curve.

The radial width *W*_r_ of the spot on the 2D multidetector measured at the peak of the rocking curve (Fig. 2a) is given by

6 $$W_{\mathrm{r}}^2 = \frac{{a^2c^2 + a^2\sigma ^2 + 4\sigma ^2c^2}}{{a^2 + c^2 + \sigma ^2}}$$

Equations (5) and (6) may be solved simultaneously to find the unknowns *σ* and *c*, and therefore *γ*. However, due to the smallness of *θ*_B_, it is difficult to quantify *γ* without significant uncertainty. As an alternative, in Fig. 4d we calculate *W*_r_ assuming *γ* = 0 and with *σ* determined from *W*_*ω*_. The *W*_r_ values calculated reproduce the measurements for fields up to 0.27 T at 1.6 K (Fig. 4d), indicating that $\gamma \ll {\mathrm{\Delta }}\lambda {\mathrm{/}}\lambda = 0.1$ in this field range. At higher fields the measured values diverge from the *γ* = 0 line. This indicates that *γ* must increase to the order of Δ*λ*/*λ* = 0.1, i.e. the autocorrelation length parallel to **g** shrinks to ≈ 3*a*_0_.

### Reverse Monte Carlo refinement

In reverse Monte Carlo (RMC) refinement, the in-plane positions of vortices are simulated on a computer. These positions are recursively modified using a Monte Carlo procedure with cost function $\chi ^2 = {\sum} {\kern 1pt} \left( {R_{{\mathrm{sim}}}(\omega ) - R_{{\mathrm{exp}}}(\omega )} \right)^2{\mathrm{/}}\delta _{{\mathrm{exp}}}(\omega )^2$ where *R*_exp_(*ω*) is the experimentally determined angular dependence of the rocking curve measured in the perpendicular geometry, with corresponding uncertainty *δ*_exp_(*ω*). Simulated rocking curves *R*_sim_(*ω*) are calculated by convolving the elastic structure factor with the experimental resolution before scaling by a factor *F*, chosen to minimise *χ*^2^. Simulated vortex ensembles are chosen to be sufficiently large that finite size effects are insignificant compared to the experimental *R*_exp_(*ω*) widths, varying from 40,000 vortices used for the 0.17 T, 2.7 K dataset (intrinsic simulation width *w*_s_ = 0.29°) to 220,000 vortices used for the 0.13 T, 1.6 K dataset (*w*_s_ = 0.12°). Our implementation here extends that used previously for niobium^19^ by incorporating a simulated annealing procedure to assure a global minimum in *χ*^2^ and to speed up the refinement. Once the minimum in *χ*^2^ is reached, in-plane vortex-vortex correlation functions are calculated directly from the simulated ensemble. The simulated ensemble is not unique. For example, it is possible that the measured rocking curve at 0.17 T, 2.7 K (Fig. 7d) could also be reproduced by an ensemble containing large ($\gg 30a_0$) domains in which the Bragg glass regime persists, but with average bond angles of neighbouring domains differing to reproduce the observed 3.5° wide distribution in *ω*. This scenario, for which we note that fracturing of the vortex lattice still occurs, is not accessible in our RMC refinements as the exceptionally large ensemble sizes required lie beyond the computational resources currently available. Similarly, the extra degree of freedom realised by *F* leads to a gradual decay of correlations with increasing simulation time even after *χ*^2^ is minimised^19^, so many different vortex ensembles are generated in each RMC run that all reproduce the shape of the experimental rocking curve. These ensembles are observed to yield the same form for each correlation function^19^, though the ordinate scale should not be taken too seriously. The ensembles obtained here are all found to be essentially free of dislocations: there is no need to impose minimum nearest neighbour distances or planarity constraints^19^.

### Data availability

The data that support the findings of this study are available from the corresponding author upon request.
